# Changes in intraocular pressure and optic nerve sheath diameter in patients undergoing robotic-assisted laparoscopic prostatectomy in steep 45° Trendelenburg position

**DOI:** 10.1186/s12871-017-0333-3

**Published:** 2017-03-11

**Authors:** Sebastian Blecha, Marion Harth, Felix Schlachetzki, Florian Zeman, Christiane Blecha, Pierre Flora, Maximilian Burger, Stefan Denzinger, Bernhard M. Graf, Horst Helbig, Michael T. Pawlik

**Affiliations:** 1 Department of Anaesthesiology, University Medical Centre Regensburg, Franz-Josef-Strauss-Allee 11, 93053 Regensburg, Germany; 2 Department of Anaesthesiology, Caritas St. Josef Medical Center, University of Regensburg, Landshuter Str. 65, 93053 Regensburg, Germany; 3 Department of Neurology, University of Regensburg, and Clinic for Neurological Rehabilitation II, medbo Bezirksklinikum Regensburg, Universitätsstraße 84, 93053 Regensburg, Germany; 4 Centre for Clinical Studies, University Medical Centre Regensburg, Franz-Josef-Strauss-Allee 11, 93053 Regensburg, Germany; 5 Department of Ophthalmology, University Medical Centre Regensburg, Franz-Josef-Strauss-Allee 11, 93053 Regensburg, Germany; 6 Department of Urology, Caritas St. Josef Medical Center, University of Regensburg, Landshuter Str. 65, 93053 Regensburg, Germany

**Keywords:** Intraocular pressure, Optic nerve sheath diameter, Robotic-assisted laparoscopic prostatectomy, steep Trendelenburg position

## Abstract

**Background:**

To evaluate changes in intraocular pressure (IOP) and intracerebral pressure (ICP) reflected by the optic nerve sheath diameter (ONSD) in patients undergoing robotic-assisted laparoscopic prostatectomy (RALP) in permanent 45° steep Trendelenburg position (STP).

**Methods:**

Fifty-one patients undergoing RALP under a standardised anaesthesia. IOP was perioperatively measured in awake patients (T0) and IOP and ONSD 20 min after induction of anaesthesia (T1), after insufflation of the abdomen in supine position (T2), after 30 min in STP (T3), when controlling Santorini’s plexus in STP (T4) and before awakening while supine (T5). We investigated the influence of respiratory and circulatory parameters as well as patient-specific and time-dependent factors on IOP and ONSD.

**Results:**

Average IOP values (mmHg) were T0 = 19.9, T1 = 15.9, T2 = 20.1, T3 = 30.7, T4 = 33.9 and T5 = 21.8. IOP was 14.0 ± 7.47 mmHg (mean ± SD) higher at T4 than T0 (*p* = 0.013). Univariate mixed effects models showed peak inspiratory pressure (PIP) and mean arterial blood pressure (MAP) to be significant predictors for IOP increase. Mean ONSD values (mm) were T1 = 5.88, T2 = 6.08, T3 = 6.07, T4 = 6.04 and T5 = 5.96. The ONSD remained permanently >6.0 mm during RALP. Patients aged <63 years showed a 0.21 mm wider ONSD on average (*p* = 0.017) and greater variations in diameter than older patients.

**Conclusions:**

The combination of STP and capnoperitoneum during RALP has a pronounced influence on IOP and, to a lesser degree, on ICP. IOP is directly correlated with increasing PIP and MAP. IOP doubled and the ONSD rose to values indicating increased intracranial pressure. Differences in the ONSD were age-related, showing higher output values as well as better autoregulation and compliance in STP for patients aged <63 years. Despite several ocular changes during RALP, visual function was not significantly impaired postoperatively.

**Trial registration:**

Z-2014-0387-6. Registered 8 July 2014.

## Background

Prostate cancer is the most common cancer in men (31.9%) and the third leading cause of cancer death (11%) in Germany [1]. Depending on the tumour entity, prostate resection is one treatment option. Robotic-assisted laparoscopic prostatectomy (RALP) is a popular and increasingly used alternative to open prostatectomy because of benefits such as minimal invasion, better short-term outcome and improved functional results [2, 3]. Actually the RALP seems to be at least equal or even superior in oncologic efficacy and complication rates compared to open prostatectomy and in 2016 any small incremental expense justifies its use [4, 5]. RALP is one of the most technically advanced treatment modalities, and its advantages include stereoscopic visualization and good manoeuvrability with 7 envelopes in the operating area. RALP requires a steep (23° to 35°) Trendelenburg position (STP) and a CO2 pneumoperitoneum. STP may lead to pathophysiological changes such as pulmonary dysfunction with formation of atelectasis and increasing airway pressure as well as ocular complications [6]. In 2007, Weber et al. [7] firstly reported 2 patients with bilateral ischaemic optic neuropathy (ION) after a da Vinci robotic-assisted procedure. Lee [8] reported 3 patients with postoperative visual loss (POVL) after RALP, 2 of them with bilateral POVL (67%). These cases were documented in the Registry of the American Society of Anaesthesiologists (POVL) between 2006 and 2010. STP has raised concerns that prolonged elevation of venous pressure in the head may increase the risk of developing ION. However, no investigations have yet been made into intraoperative changes in intraocular pressure (IOP) and optic nerve sheath diameter (ONSD) − correlating with intracranial pressure (ICP) − and their adverse ocular effects. Almost all studies were performed by doing a 30° up to a 35° Trendelenburg position. The operative conditions get better the steeper the positioning, providing excellent intraabdominal view and probably less bleeding. The hypothesis of the study is that patients placed in steep Trendelenburg position for several hours have a high risk for ocular changes and peri- and postoperative complications.

The aim of this study was to investigate the influence of capnoperitoneum and permanent 45° STP on IOP and ONSD in patients undergoing RALP. We also analysed the influence of age, body mass index (BMI), peak inspiratory pressure (PIP), mean arterial blood pressure (MAP), duration of surgery and STP on IOP and ONSD during RALP. Perioperative and postoperative complications were also recorded.

## Methods

This single-centre, prospective and nonrandomised study was approved by the local institutional review board (Protocol no. 14-101-0107) and registered at the local Centre for Clinical Studies (Z-2014-0387-6. Registered 8 July 2014). Informed consent was obtained from 51 patients scheduled for elective prostatectomy at the Department of Urology in Regensburg. All patients were recruited between January 2015 and August 2015. Main exclusion criteria were pre-existing eye disease (diabetic retinopathy, glaucoma and retinal detachment), history of eye surgery, age >80 years, BMI >40, American Society of Anaesthesia (ASA) physical status > III, known cardiac insufficiency and pulmonary hypertension.

### Anaesthesia protocol and surgical technique

**Fig. 1:**
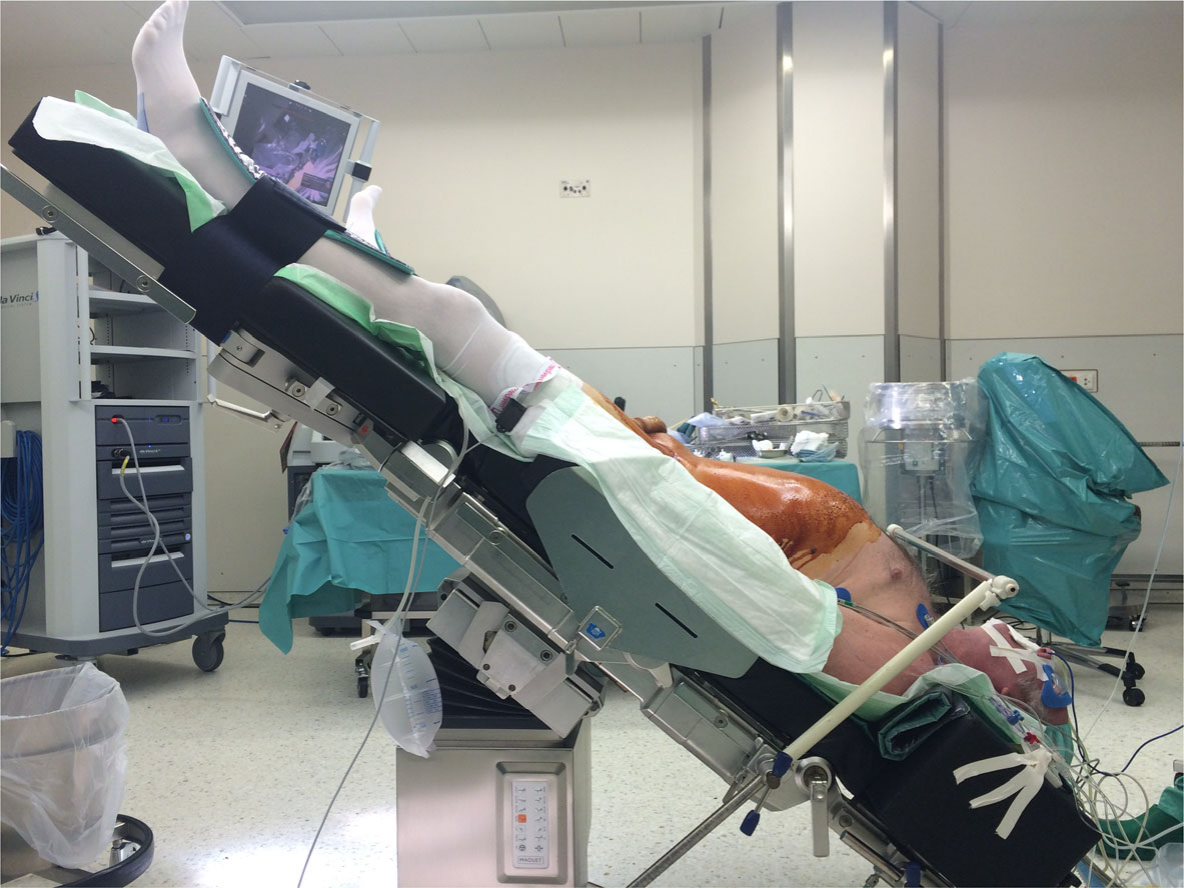
Preparation and test of 45° Trendelenburg position before start of RALP

The anaesthesia protocol was standardised for drugs used during RALP and exclusively conducted by the same 2 anaesthesiologists throughout the entire study. After baseline IOP measurement, patients received 2 mg of midazolam for premedication and 0.2 mg of piritramid per kilogramme (kg) body weight for the placement of a PiCCO radial artery catheter (PULSION Medical Systems SE, Germany) for invasive blood pressure measurement under local anaesthesia. Anaesthesia was induced with propofol (2–3 mg/kg), remifentanil (1.5 μg/kg bolus and continuous application of 0.3 μg/kg/min) and rocuronium (0.5 mg/kg). After tracheal intubation, anaesthesia was continued with propofol as total intravenous anaesthesia guided by the Bispectral Index™ (BIS Vista Monitor, Aspect Medical, Germany) between 40 and 50; remifentanil was reduced to 0.2 μg/kg/min. Neuromuscular transmission was monitored with a peripheral nerve stimulator to maintain one twitch of the train-of-four (TOF). Relaxation with rocuronium was repeated in the case of TOF >1/4 and finished 45 min before the end of the procedure. Arterial blood pressure was kept stable with a maximum decrease of 20% of its pre-induction value. Controlled ventilation maintained end tidal CO2 (etCO2) between 30 and 40 mmHg. Pneumoperitoneum was created by intraperitoneal insufflation of CO2 with the patient in supine position. All patients were then placed in STP (45° from horizontal), which is the maximal Trendelenburg angle of the Maquet surgical table (Maquet®, MAQUET Vertrieb und Service Deutschland GmbH, Germany) (Fig. 1). The 45° STP as the standard in our hospital optimised the view for the surgeon and minimised blood loss. Throughout surgery, intraabdominal pressure was maintained at 15 mmHg using CO2 for insufflation. Only during preparation of Santorini’s plexus was the intraabdominal pressure increased up to 25 mmHg to reduce venous bleeding. Surgery was exclusively conducted by the same highly experienced urologist. Crystalloid fluid was limited to a maximum of 1000 ml before terminating vesico-urethral anastomosis.

### Measurements of intraocular parameters and the optic nerve sheath diameter

**Fig. 2:**
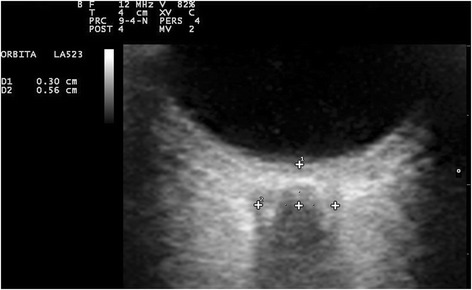
Measurement of the optic nerve sheath diameter

IOP was measured in each patient in both eyes with an Icare® PRO rebound tonometer (Icare Finland Oy, Finland), which does not require any topical anaesthesia of the cornea. The tonometer was calibrated according to the manufacturer’s guidelines before each application. IOP was bilaterally measured in each patient at 6 predefined time points (Table 1). The ONSD in both eyes (time points T1 to T5) was measured in anaesthesia with the Esaote MyLab™One/Touch portable ultrasound by 1 of 2 trained anaesthesiologists and verified by an expert for ONSD ultrasound examination by means of a planar ultrasonic probe with a frequency of 12 MHz. Mechanical index, focal positioning and reduction of B-mode gain was adjusted as previously described elsewhere [9, 10]. During the examination, the patient laid supine with eyes closed and a layer of acoustic gel applied to the closed eyelids. Pressure on the globe was as light as possible to avoid a decrease in blood flow velocity in the retroorbital vessels [11]. The optic nerve appears as a hypoechogenic structure beyond the retina and optic disc, providing an anatomical landmark for ultrasound examination. The hyperechogenic structure around the optic nerve represents the subarachnoid space, which is bordered by the hypoechoic dura mater. The ONSD was measured 3 mm behind the optic disc by determining the distance between the medial hypoechogenic borders of the ONS (Fig. 2). Up to 3 measurements were recorded in each eye at T1 to T5, and the median of all measurements was taken. Next to a low mechanical index, all examinations lasted less than 5 min for each eye to further avoid thermal and cavitation damage.

**Table 1:** Time points of IOP and ONSD measurements

Time points of measurements		IOP	ONSD
T0	Patient awake in supine position before induction of anaesthesia	X	-
T1	20 min after induction of general anaesthesia in supine position	X	X
T2	After insufflation of the abdomen with CO_2_ in supine position	X	X
T3	After 30 min in 45° Trendelenburg position with the abdomen still insufflated with CO_2_	X	X
T4	Control of Santorini’s plexus in 45° Trendelenburg position with CO_2_ still insufflated	X	X
T5	Anaesthetised before awakening in supine position	X	X

### Registration of postoperative (visual) complications

Postoperative all patients were observed in the recovery room, 8 h later on the ward and the next day and received a clinically visual and perimetric examination.

### Statistical analysis

Due to the exploratory nature of this study and the lack of a primary endpoint with expectable effect sizes, no sample size calculation was performed a priori. To get robust effects in a feasible amount of time, we decided to include about 50 patients into this study. To analyse the influence of the variables age, BMI, intravenous infusion, PIP and MAP on IOP and ONSD, linear mixed models were used. These models account for correlated measurements within each patient because of several time points. Each variable was analysed in a separate model, containing time as an additional factor. The covariance structure between the time points was set to autoregressive. Effect estimates of significant variables are presented as slopes (effect on IOP and ONSD per one unit change of the variable) with corresponding 95% confidence intervals. All reported *p* values are two-sided, and a *p* value of 0.05 is considered the threshold of statistical significance. Because of the explorative nature of this study, no adjustment for multiple testing was done. Data were analysed with the software SAS 9.4 (SAS Institute Inc., Cary NC).

## Results

Fifty-one male patients were enrolled in this study. We registered IOP and ONSD for 102 eyes of 51 patients. Patient characteristics and common surgical variables are shown in Table 2. ASA classification was I for 5 patients (9.8%), II for 38 patients (74.5%) and III for 8 patients (15.7%).

**Table 2:** Patient demographics and operative variables

Variable	Median (range)
Age	63.7 (49–77)
BMI (kg/m^2^)	28.4 (20.3–38.4)
Duration of surgery (min)	218 (120–357)
Duration of 45° Trendelenburg position (min)	198 (109–331)
Intravenous infusion during operation (ml)	880 (550–2200)

### Individual and surgical influences on IOP during RALP

**Fig. 3:**
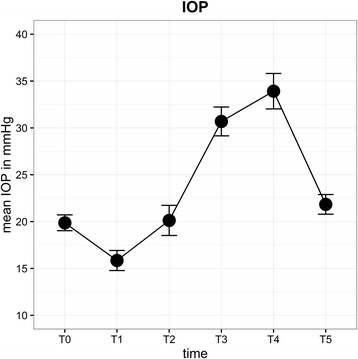
Mean IOP for each time point (±SD)

**Fig. 4:**
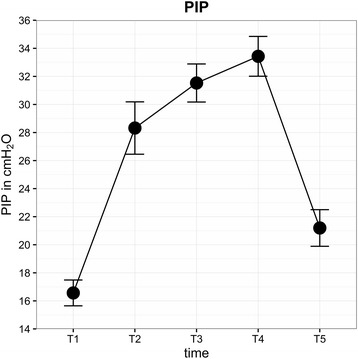
Mean PIP for each time point (±SD)

**Fig. 5:**
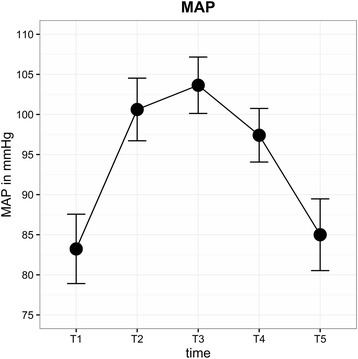
Mean MAP for each time point (±SD)

Average IOP (± SD) of all eyes (mmHg) for each time point was as follows: T0 = 19.9 (±3.6), T1 = 15.9 (±4.8), T2 = 20.1 (±6.6), T3 = 30.7 (±6.3), T4 = 33.9 (±7.4) and T5 = 21.8 (±4.3) (Fig. 3). IOP after induction of anaesthesia (T1) was significantly lower than IOP at T0 (*P* < 0.0001). In contrast, IOP at T3 (*P* = 0.008) and T4 (*P* = 0.013) was significantly higher than at T0. IOP was highest at STP (T4). In 14% of patients, IOP at T4 was higher than 40 mmHg, and the highest IOP measured was 59.6 mmHg. BMI, duration of surgery and STP did not influence IOP. A linear relationship between PIP and IOP was observed: IOP rose significantly with increasing airway peak pressure (effect estimate 0.17 (95%-CI: 0.03, 0.31), *P* = 0.018). PIP in STP reached levels above 30 cmH2O most of the time (Fig. 4). MAP increased after induction of anaesthesia with the start of capnoperitoneum and was significantly higher during STP (Fig. 5). A direct correlation was found between IOP and MAP. Increasing MAP correlated with significantly increased IOP in STP (effect estimate 0.08 (95%-CI: 0.04, 0.13), *P* < 0.001).

### Individual and operational influences on ONSD during RALP

**Fig. 6:**
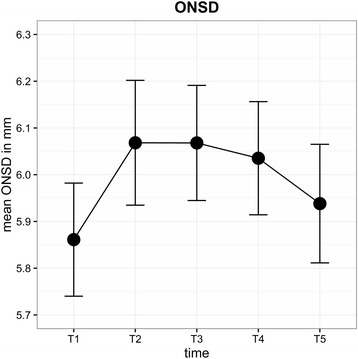
Mean ONSD for each time point all patients (±SD)

**Fig. 7:**
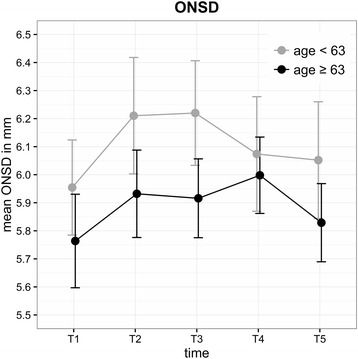
Age differences of changes in the ONSD (±SD)

The mean (± SD) ONSD (mm) for each time point was as follows: T1 = 5.88 (±0.44), T2 = 6.08 (±0.52), T3 = 6.07 (±0.49), T4 = 6.04 (±0.50) and T5 = 5.96 (±0.49). The ONSD increased by about 0.2 mm (3.4%) after the start of capnoperitoneum (T2). During STP, the ONSD remained increased and was higher at the end of surgery (T5) than at the initial level (Fig. 6). With respect to ONSD, the observed changes did not influence BMI, PIP, MAP, duration of surgery or STP. The differences in the changes in the ONSD during RALP were age-related. Interestingly, the ONSD was on average 0.21 mm (95%-CI: 0.04, 0.38) wider (*P* = 0.017) in patients aged <63 years (median age) and showed greater variations in diameter than in older patients (Fig. 7). During prolonged STP (T4), the ONSD decreased in patients aged <63 years but increased in older patients.

### Observed complications

Neither ocular nor neurological complications were observed in the recovery room, 8 h later on the ward and the next day. One patient experienced perioperative cardiac ischemia; reporting angina symptoms in the recovery room he received postoperative coronary angiography with stent implantation due to significant coronary stenosis.

## Discussion

In this study, we examined the changes in IOP and ONSD between supine and STP during RALP. The combination of STP and capnoperitoneum during RALP has a pronounced influence on IOP and, to a lesser degree, on ICP. IOP was directly correlated with increasing ventilation pressure and MAP. However, no ocular or cerebral pressure-related complications occurred in our study population.

### Changes in IOP during RALP

The mean baseline IOP of our awake patients was 19.9 mmHg; thus, it was slightly higher than in other studies (18.0 mmHg, 14.9 mmHg), which may depend on the fact that we measured IOP with a rebound tonometer without topical anesthesia in the awake patient [12, 13]. Induction of anaesthesia in supine position decreased the mean IOP from 19.9 to 15.9 mmHg. The ocular hypotensive effect of anaesthetics has been known for many years and may counteract IOP increases induced by RALP [14]. IOP is significantly more reduced by propofol than by volatile anaesthetics [15, 16]. During RALP, the mean IOP more than doubled between T1 and T4 (15.9 to 33.9 mmHg) in this study, and the highest IOP value measured was 59.6 mmHg. Awad et al. [17] also reported that the mean IOP was 5 mmHg lower after induction of anaesthesia and had increased almost threefold (10.7 to 29.0 mmHg) in STP at the end of surgery. This mean was on average 13 mmHg higher than pre-anaesthesic values. In another study, the mean IOP had increased after induction of anaesthesia from 9.8 mmHg to 24.2 mmHg at the end of STP [12].

Molloy found that baseline IOP and duration of surgery were the only factors predicting increased IOP [18]. In our study, age, BMI, duration of surgery and STP did not affect IOP, but MAP and PIP showed a direct relationship and significant effect on IOP. However, respiration in terms of lung protective ventilation or hypotension is hardly feasible when using STP. One way of reducing IOP could be a variation in the Trendelenburg position to avoid extreme IOP increases. Raz et al. applied a modified Z-TP during RALP and found a significant positive effect on patient neuro-ocular safety by lowering intraocular pressure without any negative effects on surgery. Increased IOP can also be reduced by drugs [19]. In a study by Molloy et al., 32.5% of patients undergoing lengthy laparoscopic surgery in STP had an IOP higher than 40 mmHg [20]. Treatment with dorzolamide-timolol eye drops significantly reduced elevated IOP.

On the basis of this study, we were able to quantify the changes in IOP throughout the procedure. We therefore concluded that patients treated with robotic prostatectomy reach IOP levels comparable to those observed in patients with glaucoma. However, possible adverse ocular effects as a consequence of IOP changes have not yet been investigated. In our study, no patient experienced ischaemic optic neuropathy after RALP clinically represented by scotoma or significant visual loss.

### Changes in the ONSD during RALP

In this study, the ONSD did not significantly exceed the initial value (maximum rise 3.4%, 0.2 mm). Another study reported that the ONSD increased by 12.5% (0.6 mm) during CO2 pneumoperitoneum and STP for 20 patients undergoing RALP [21]. Chin et al. also found a significant increase in the ONSD (0.6 mm) between supine and STP in 21 patients [22]. The basic value of the ONSD in this study was 5.88 mm. In their review, Soldatos et al. [23] determined 5.7–6.0 mm as the cut-off value of the ONSD that provides the best accuracy for predicting intracranial hypertension (ICP >20 mmHg). For patients with severe brain injury (subarachnoid haemorrhage, intracranial hematoma or stroke) was showed that an ONSD threshold of 5.2 mm or 5.86 mm as a predictor for ICP >20 mmHg proved to be an attractive combination of sensitivity (94 and 95%) and specificity (76 and 79%) [24, 25]. In the aforementioned studies by Kim [21] and Chin [22], the initial values for the ONSD were 4.5 and 4.8 mm, which may indicate a difference between Korean and European patient populations. Another reason for the increased initial value for the ONSD in our study could be PEEP-triggered ventilation. PEEP increases intra-thoracic pressure. The venous backflow from the brain resulting in increased ICP may explain the above average baseline value. In general, ONSD changes depend on individual patient characteristics and body position. Fichtner et al. reported an ONSD reduction of about 0.53 mm from supine to upright position for patients with orthostatic headache [26]. During RALP, the ONSD rose above 6.0 mm, which suggests elevated ICP (Fig. 6). In addition, Whiteley et al. found a direct correlation between increased ONSD values and MAP [27]. We did not observe any influence of MAP in our study. The non-significant increase in the ONSD may be due to the effect of PEEP and non-linearity of ONSD elasticity [28]. The impact of a slight increased ONS remains after RALP unclear and is probably not of clinical importance.

However, we found an age-related difference (median 63 years) in the changes in the ONSD during RALP. Younger patients had a significantly higher baseline ONSD under mechanical ventilation, suggesting higher elasticity of the dura mater. Over the course of STP (T3 to T4), the ONSD decreased in younger patients, which may indicate better adaptability of the ONSD or intracranial pressure, respectively (Fig. 7).

### Limitations of the study

Because of logistic preoperative constraints, we did not measure the ONSD prior to intubation. Neither ocular nor neurological complications were observed in our defined cohort. Larger prospective studies with RALP are required to further evaluate the relationship between STP and permanent ocular changes and to make recommendations regarding the prevention and treatment of increased intraoperative IOP.

## Conclusion

In conclusion, we found that IOP and ONSD increased significantly in a time-dependent way in patients undergoing RALP in 45 ° STP. IOP doubled and the ONSD rose to values indicating slight increased intracranial pressure, but no patient reported visual impairment or at worst POVL. We observed age-related differences in the ONSD with a higher initial value and better autoregulation in STP for younger patients. RALP is associated with significant measurable ocular changes, namely an increase of IOP and ONSD.

## Abbreviations

ASA: American Society of Anaesthesia
BMI: Body mass index
ICP: Intracerebral pressure
ION: Ischaemic optic neuropathy
IOP: Intraocular pressure
MAP: Mean arterial blood pressure
ONS: Optic nerve sheath
ONSD: Optic nerve sheath diameter
PEEP: Positive end-expiratory pressure
PIP: Peak inspiratory pressure
POVL: Postoperative visual loss
RALP: Robotic-assisted laparoscopic prostatectomy
STP: Steep Trendelenburg position
TOF: Train-of-four
